# The function of S100A4 in pulmonary disease: A review

**DOI:** 10.1097/MD.0000000000033466

**Published:** 2022-04-07

**Authors:** Ting Wang

**Affiliations:** a Department of Respiratory Medicine, Xi’an People’s Hospital (Xi’an No. 4 Hospital), Xi’an, China.

**Keywords:** asthma, chronic obstructive pulmonary disease, idiopathic pulmonary fibrosis, lung cancer, S100A4

## Abstract

S100 protein family, which represents 25 relatively small calcium binding proteins, is involved in many intracellular and/or extracellular processes, including differentiation, apoptosis, migration/invasion, Ca^2+^ homeostasis, inflammation, and tissue repair. As an important member, S100A4 was reported to have an abnormal expression in several lung diseases, such as lung cancer, pulmonary hypertension, idiopathic pulmonary fibrosis (IPF), etc. For example, in lung cancer, S100A4 was demonstrated to be associated to metastatic tumor progression and epithelial to mesenchymal transition (EMT). In IPF, S100A4 was considered as a promising serum biomarker predicting disease progression. Various studies in recent years focused on the S100A4 function in lung diseases, showing researchers’ interests on this protein. It is necessary to focuses on relative studies, and make a comprehensive understanding of S100A4 in common pulmonary diseases. By doing this, this paper provides a review of the evidence for S100A4 in lung cancer, chronic obstructive pulmonary disease (COPD), asthma, IPF and pulmonary hypertension.

## 1. Introduction

The S100 protein family, which was discovered firstly by Moore et al in 1965, have been proved to have 25 small acidic Ca^2+^ combined with cytotoxic proteins.^[1]^ Two founding members, S100A1 and S100B, were found to be soluble in 100% saturated ammonium sulfate at that time, thus this family was named S100.^[2,3]^ As a largest one in the EF-hand superfamily, S100 proteins implicated in multiple intracellular and/or extracellular regulatory activities. According to the roles in Calcium regulation, S100 members could be divided into 2 groups.^[4]^ The first group gets involved in translating the signals via detecting the Ca^2+^ ions levels by calcium sensors. The second one, which consists of Ca^2+^ buffer, could bind free cell cytoplasm Ca^2+^ ions and modulate the calcium signals.^[5]^ Thus, these second messenger meditators participate in the control of an array of cellular processes ranging from muscle contraction to cell behaviors. In a variety of diseases, S100s have been reported to have an abnormal expression. S100A2, for example, was firstly found to act as a tumor suppressor gene, having a downregulated expression in skin, lung, kidney and prostate tumors.^[6–8]^ On the other hand, S100A2 was found to have an upregulation in some cancer types as well, including pancreatic cancer, gastric cancer and epithelial ovarian cancer. S100A6 has an elevated expression in the serum of gastric cancer patients^[9]^; S100A7 levels have been found to be augmented in cerebrospinal fluid of patients with Alzheimer disease^[10]^; blood levels of S100A8/9 were reported to be increased in obesity and coronary artery diseases^[11]^; S100A12 has been reported to have an enhanced expression in inflammatory diseases and diabetes.^[12]^ In pulmonary diseases, such as asthma, chronic obstructive pulmonary disease (COPD), idiopathic pulmonary fibrosis (IPF), cystic fibrosis, pulmonary hypertension, and lung cancer, S100 family members have also been observed to have dysregulated responses. A quantity of studies in recent years were undertaken to identify the exact role of this family in pulmonary disease pathogenesis and therapy. Of all the S100 members, S100A4 is the most extensively studied, and has been given many names, such as PEL-98, 18A2, 42A, CAPL, P9KA, metastasin (MTS-1), etc.^[13]^ The human S100A4 gene is located in the epidermal differentiation complex on chromosome 1q21, which is prone to chromosomal rearrangements, thus the encoded protein is involved in many physiological functions, including cell proliferation, invasion and metastasis.^[14]^ Meanwhile, its function in multiple pulmonary diseases has been confirmed.^[15]^ In this review, we summarize the evidence concerning S100A4 and pulmonary diseases and discuss the mechanisms through which S100A4 plays its diverse functions in common lung diseases.

## 2. Lung cancer

**Figure 1.**
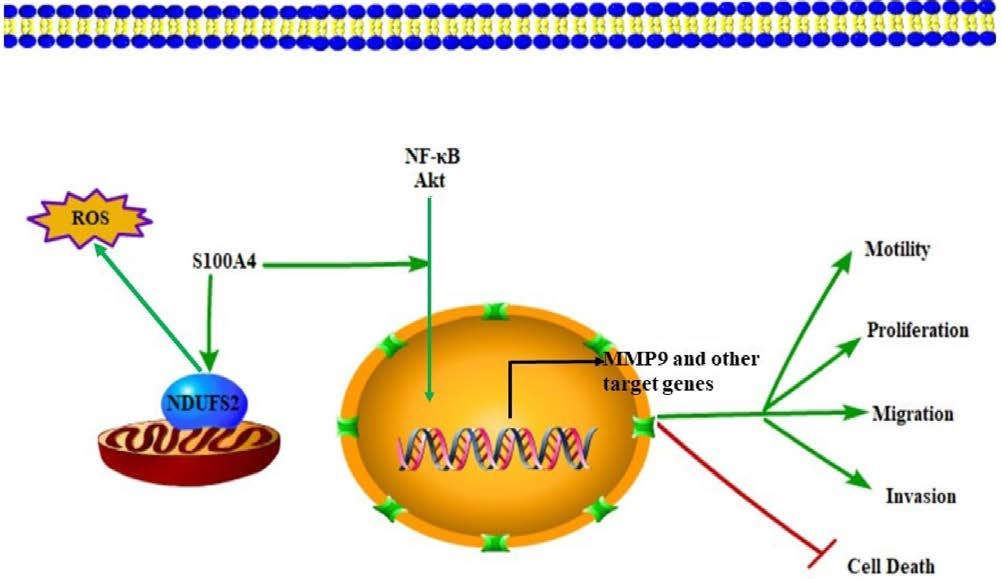
S100A4 proteins get involved in NF-kB and AKT signaling, affecting the cell activities.

S100A4 was firstly reported to have an association with metastatic tumor in 1989. Ebralidze MS et al detected the expression of mts1 in the metastatic phenotype of different transformed and normal cells using Northern blot analysis, and results showed that the overexpression of mts1was associated with a high degree of metastasis.^[16]^ Relative researches later on found that the high level of S100A4 implicates in metastasis and progression of various malignancies; it could also be used as a marker in epithelial to mesenchymal transition (EMT).^[17–19]^ In lung cancer, Takenaga K et al firstly demonstrated that pEL98 (mts1) expression was related to invasive and motile abilities in clones derived from lung carcinoma, and they drew a conclusion that pEL98 (mts1) had a function in regulating cell invasiveness and tumor cell motility.^[20]^ Their later research found cell motility and in vitro invasiveness were suppresses in the antisense S100A4 RNA-expressing Lewis lung carcinoma, supporting the above conclusion from the reverse side.^[21]^ Compared with leading-edge peripheral parts, S100A4, along with other mesenchymal markers, had a lower expression in the central region of non-small cell lung cancer. Moreover, its high expression was proved to be related to poor differentiation and advanced stage of adeno- and squamous cell carcinoma. Concerning of the exact mechanism of S100A4 in lung cancer, Stewart RL et al demonstrated the knockdown of S100A4 could inhibit NF-κB activity and decreased TNFα-induced MMP9 expression (Fig. 1), indicating these downstream molecules in its regulation of lung cancer cellular biological activities.^[22]^ A recent study found that the overexpression of S100A4 could also enhance cell proliferation and inhibit starvation-induced autophagy via the Wnt/β-catenin signaling pathway in A549 cells.^[23]^ In another study of NSCLC cell lines, S100A4 was also proved to up-regulate mitochondrial complex I subunit NADH dehydrogenase (ubiquinone) Fe-S protein 2, thus promoting cells’ invasion and altering metabolism (Fig. 1).^[24]^ Researches focusing on the molecules that regulate S100A4, on the other hand, found that plakoglobin, a tumor/metastasis suppressor, restored its tumor suppressor activity in NSCLC cells by upregulating S100A4 with P53 mutants (H1299).^[25]^ In another research, a long noncoding RNA (linc01833) was proved to adsorb miR-519e-3p through a sponge and regulate S100A4 in lung adenocarcinoma progression.^[26]^

## 3. Chronic obstructive pulmonary disease

COPD is characterized with chronic airway inflammation and progressive fixed airflow limitation, usually having an association with tobacco smoke inhalation.^[27]^ An explanation for airway remodeling in chronic inflammatory respiratory diseases, such as COPD, is involving with the differentiation of airway epithelial cells to a mesenchymal phenotype, which is also named EMT.^[28]^ In 2010, researchers firstly examined EMT markers, including S100A4, in airway biopsy tissue from subjects with COPD.^[29]^ Compared with control groups, obviously increased reticular basement membrane cell S100A4 staining could be seen in smokers with COPD. Their later studies in 2011 and 2014 found that S100A4 had a significantly high expression in basal epithelium compared to infiltrating macrophages, fibroblasts, as well as immune cells, and S100A4 positive cell numbers sharply decreased after the treatment of inhaled fluticasone propionate, providing additional support for active EMT and anti-EMT effects of inhaled corticosteroids in COPD.^[30,31]^ Further study conducted by Mahmood MQ et al showed S100A4 tended to have an increased expression in small airway, compared to large airways, of subjects with chronic airflow limitation.^[32]^ An elevated expression of S100A4 could also be found in serum, tissues and vasculature of patients with COPD. Reimann S et al performed real-time RT-PCR analysis and immunohistochemistry to investigate the expression of S100A4 in laser-microdissected intrapulmonary arteries of COPD patients, and data revealed S100A4 had an enhanced expression at both levels.^[33]^ These findings were mirrored by Enzyme-Linked Immunosorbent Assay analysis of S100A4 in the serum of patients with COPD.^[34]^ Moreover, serum S100A4 was inversely related to pulmonary function among COPD patients. Concerning of the mechanism, the study of Jiang B et al demonstrated that exposure of the epithelium to cigarette smoke extract and exposure of the mice to cigarette smoke can induce EMT by activating the Akt signaling pathway, suggesting this pathway could be a regulator for S100A4 in COPD (Fig. 1).^[35]^

## 4. Asthma

Asthma, which is one of the most common forms of respiratory disease, is characterized by immune hyper-responsiveness, airway inflammation, eosinophilic infiltration and mucus hypersecretion.^[36,37]^ It has been demonstrated that damage-associated molecular pattern can activate the innate immune response through interacting with pattern recognition receptors. S100A4 could act as damage-associated molecular pattern once secreted by the action of specific chemokines, stress or necrosis.^[38]^ Related studies found that S100A4 had an elevated levels in several inflammatory diseases such as arthritis, kidney fibrosis and neuronal injury.^[39,40]^ In chondrocytes, stimulation of T cells with S100A4 was proved to have an increased production of cytokines, especially eotaxin-2 and granulocyte colony-stimulating factor, both of which are important factors in the pathogenesis of asthma. In 2018, Huang X et al tested the expression in induced sputum and plasma from asthmatics and healthy controls. Data showed that S100A4 was overexpressed in the sputum rather than in plasma in asthmatics, and its high expression was associated negatively with some lung function parameters and were correlated positively with sputum lymphocyte and eosinophilia. In the asthma mouse model, the expression of S100A4 was also significantly higher in the lung as well as in BALF. Further study showed that LY294002, a PI3K inhibitor, could decrease S100A4 in both lung and BLAF markedly in asthmatic mice.^[41]^ In mast cell, S100A4 gene deficiency was identified to dampen its activation both in vitro and in vivo, suggesting S100A4 may participate in the regulation of allergic responses through regulating the activation of mast cells. Furthermore, the airway remodeling in the chronic asthma model was confirmed to be attenuated by inhibition of soluble epoxide hydrolase, dapagliflozin and ZDHXB-101 (3’,5-Diallyl-2, 4’-dihydroxy-[1,1’-biphen-yl]-3,5’-dicarbaldehyde), and the expression of remodeling-related molecular markers reduced, including S100A4.^[42–44]^ An in-depth study showed that the synthesis and secretion of S100A4 in airway smooth muscle tissues could be stimulated by inflammatory mediators and that extracellular S100A4 acted via RAGE to mediate airway smooth muscle inflammation.^[45]^

## 5. Idiopathic pulmonary fibrosis

IPF is a progressive and lethal fibrotic lung disease characterized by alveolar epithelial cell injury and activation, formation of myofibroblast foci, and exaggerated extracellular matrix accumulation in the lung parenchyma.^[46,47]^ Patients are often behaved progressive dyspnea and restrictive physiology on pulmonary function testing.^[48]^ In 2010, Degryse AL et al developed lung fibrosis mice model by intratracheal bleomycin. Data showed that 50% of S100A4 + lung fibroblasts were derived from epithelial mesenchymal transition in those mice models established by repetitive bleomycin, which had greater fibrosis by scoring, morphometry and collagen content, compared with 33% in the single-dose model. In bronchoalveolar lavage fluid, numbers of S100A4 + macrophages were proved to be correlated well with S100A4 protein levels and the occurrence of IPF.^[48]^ The study of Zhang W et al, data suggested that S100A4 was produced and secreted by M2 polarized alveolar macrophages.^[49]^ Moreover, in vitro, extracellular S100A4 were revealed to activate both mouse and human lung fibroblasts via upregulating α-SMA and type I collagen, during which sphingosine-1-phosphate increased.^[50]^ As for its clinical application, researchers detected S100A4 levels in the sera and tissues of patients with IPF and health controls. Results showed serum S100A4 levels were undetectable in all health controls but were detectable in 26/95 IPF cases. In the lung tissues from IPF patients, aggregation of numerous S100A4-expressing cells was found around the fibroblastic foci and mature fibrotic regions. Those patients with higher serum S100A4 levels tended to have a significantly worse prognosis than their counterparts.^[51]^ Similar conclusions were also confirmed by real-time PCR and immunoblotting in fibroblasts from IPF patients and controls.^[52]^ In another study, hyaluronan (HA) was found to present in the fibroblastic focus together with CD44-expressing fibrogenic mesenchymal progenitor cells (MPC) and that ligation of CD44 by HA triggered S100A4 nuclear translocation to support IPF MPC self-renewal, suggesting that S100A4-mediated MPC fibrogenicity in IPF was regulated by HA/CD44 axis.^[53]^

## 6. Pulmonary arterial hypertension

Pulmonary arterial hypertension (PAH), which is characterized by pulmonary vessels’ remodeling and a persistent increase in the pulmonary vascular resistance, is a complex pulmonary vasculature disease with poor prognosis.^[54,55]^ EMT was considered as a critical process in PAH etiology.^[56]^ Thus, some factors implicated in EMT, including S100A4, have been verified to be associated with PAH. A recent study conducted by Laggner M et al quantified S100A4, EGF, and EGFR in patients suffering from chronic thromboembolic pulmonary hypertension and idiopathic PAH. Data analysis revealed S100A4 tissue expression positively correlated with higher grades of Heath-Edwards histopathological lesions of idiopathic PAH-derived lung tissue, while was devoid in pulmonary thrombo-endarterectomized samples.^[57]^ In the mice models of chronic hypoxia, which is considered as a significant factor in the occurrence of pulmonary hypertension, S100A4, along with CD36 were confirmed to be positively expressed in the vascular tunica media.^[58]^ Concerning of the mechanism, Lawrie A et al investigated the codependence of 5-HT receptors and serotonin transporter in regulating S100A4/Mts1 in human pulmonary artery smooth muscle cells (hPA-SMC). They found that 5-HT elevated S100A4/Mts1 mRNA levels and increased S100A4/Mts1 PA-SMC lysates and culture media, indicating a mechanistic link between the 5-HT pathway and S100A4/Mts1 in pulmonary hypertension.^[59]^ Besides that, advanced glycation endproducts (RAGE) binding S100A4 released by activated leukocytes results in the generation of reactive oxygen species and further activation of NF-κB.^[60]^ This leads to reduced bioavailability of the labile vasodilator nitric oxide, reducing its anti-inflammatory effects and possibly compromising control of vascular tone directly. Furthermore, RAGE antagonism could also prevent migration and proliferation of PA-SMC in response to 5-HT.^[60]^ Accordingly, Farmer DG et al pointed out that S100–RAGE signaling may be of key importance in pulmonary vascular homeostasis and/or disease in a review.^[60]^

## 7. Conclusion

S100A4 was demonstrated to be involved in several lung diseases, including lung cancer, COPD, asthma, pulmonary hypertension, and IPF. With more and more studies, its mechanism of action has been constantly confirmed. In the future, this protein may be used in the diagnosis, targeted treatment, and prognosis evaluation of several pulmonary diseases.

## Author contributions

**Data curation:** Ting Wang.

Writing – original draft: Ting Wang.

Writing – review & editing: Ting Wang.
